# Long-term isolation at a low effective population size greatly reduced genetic diversity in Gulf of California fin whales

**DOI:** 10.1038/s41598-019-48700-5

**Published:** 2019-08-27

**Authors:** Vania E. Rivera-León, Jorge Urbán, Sally Mizroch, Robert L. Brownell, Tom Oosting, Wensi Hao, Per J. Palsbøll, Martine Bérubé

**Affiliations:** 1 Marine Evolution and Conservation, Groningen Institute of Evolutionary Life Sciences, University of Groningen, Nijenborgh 7, 9747 AG Groningen, The Netherlands; 2 Departamento de Ciencias Marinas y Costeras, Universidad Autónoma de Baja California Sur, Km 5.5 Carretera al Sur, 23081 La Paz, Baja California Sur Mexico; 3 Blue Sea Research PO Box 15805, Seattle, WA 98115 United States of America; 4 Southwest Fisheries Science Center, NOAA Fisheries, 34500 Highway 1, Monterey, CA 93940 United States of America; 5 Centre for Coastal Studies, 5 Holway Avenue, Provincetown, Massachusetts, 02657 United States of America

**Keywords:** Conservation biology, Population genetics, Population genetics

## Abstract

The Gulf of California, Mexico is home to many cetacean species, including a presumed resident population of fin whales, *Balaenoptera physalus*. Past studies reported very low levels of genetic diversity among Gulf of California fin whales and a significant level of genetic differentiation from con-specifics in the eastern North Pacific. The aim of the present study was to assess the degree and timing of the isolation of Gulf of California fin whales in a population genetic analysis of 18 nuclear microsatellite genotypes from 402 samples and 565 mitochondrial control region DNA sequences (including mitochondrial sequences retrieved from NCBI). The analyses revealed that the Gulf of California fin whale population was founded ~2.3 thousand years ago and has since remained at a low effective population size (~360) and isolated from the eastern North Pacific (*N*_*e*_*m* between 0.89–1.4). The low effective population size and high degree of isolation implied that Gulf of California fin whales are vulnerable to the negative effects of genetic drift, human-caused mortality and habitat change.

## Introduction

Population genetic data hold the potential to provide insights into the basis of genetic variation in natural populations^1^, which in turn is a function of past and present effective population sizes, migration rates, selection, and mutation rates. A population acquires genetic variation by mutation and immigration. Genetic variation is lost by random genetic drift resulting in higher rates of loss in small and isolated populations compared to larger, connected populations^2^. Possible consequences of low genetic variation include increased genetic load and loss of adaptive traits, which in turn can elevate short- and long-term extinction rates^3–6^. Consequently, understanding the processes that shaped contemporary levels of genetic variation may add vital insights to inform the conservation and management of local populations.

Population genetic assessments have proven particularly useful to study elusive species, such as baleen whales^7–10^. Baleen whales typically undertake long-range seasonal migrations to forage in cold nutrient-rich waters at high latitudes during the summer and winter in warm tropical waters during the breeding season^11^. Exceptions to this pattern are Omura’s (*Balaenoptera omurai*)^12^ and Bryde’s whales (*Balaenoptera brydei*)^13^, which remain at low latitudes all-year as well as bowhead whales (*Balaena mysticetus*) that are confined to polar waters^14^. A few populations among those baleen whale species that undertake seasonally migrations, do not migrate but remain in the same area year-round, such as blue whales (*Balaenoptera musculus*)^15,16^ and humpback whales (*Megaptera novaeangliae*) in the Arabian Sea^17–20^.

Baleen whales are also observed year-round in the Gulf of California, Mexico. Baleen whales are commonly observed around the Midriff Islands and in the Canal de Ballenas between the east coast of the Baja California Peninsula and Isla Angel de la Guarda^21–23^. These whales were probably first noted by, and the canal named by, the Croatian Jesuit cartographer Fernando Consag during his 1746 expedition to the Upper Gulf of California undertaken to prove that Baja California was not an island. The whales in the Canal de Ballenas were identified as fin whales as early as the late 18^th^ Century^24^ and were later “rediscovered” during the 1940 expedition on the Western Flyer in the Sea of Cortez^23,25^. Gilmore^23,26^ was the first to suggest that the fin whales in Gulf of California were all-year residents, a notion later repeated by Norris^27^. Since the 1980s, regular surveys aimed at baleen whales in the Gulf of California have been conducted in the waters off the Midriff Islands, in particular Canal de Ballenas^21,23,28–30^. Fin whales, and their vocalizations have been recorded throughout the year in the Gulf of California and appear most abundant in the Upper Gulf^21,23,31,32^.

The Gulf of California fin whales appear isolated from con-specifics in the eastern North Pacific. Fin whales have not been observed in the entrance of the Gulf of California and sightings off the southern tip (south of 24°N) of the Baja California Peninsula have been scarce^31,33–35^, although some fin whales were sighted south of 24°N in February during the annual surveys between 1965 and 1967^36^. The presumed residency of this fin whale population was also supported by movement data from 11 satellite-radio tagged fin whales, which remained in the Gulf of California while transmitting location data^34,37^. Further supporting the notion of isolation were the 20 Hz fin whale calls recorded in the Gulf of California that were unique and distinct from fin whale calls recorded in the North Pacific and North Atlantic^38^. However, some occasional tempo-spatial overlap was noted in fin whale calls recorded in the Gulf of California and off Southern California, indicating the possibility of some connectivity between these two areas^39^.

Genetic analyses conducted by Bérubé *et al*.^40,41^ confirmed the presumed reproductive isolation of the Gulf of California fin whales. Bérubé *et al*.^40^ compared mitochondrial control region (mtCR) DNA sequences and genotypes at 16 nuclear microsatellite loci in 12 samples from the eastern North Pacific with 77 samples from the Gulf of California. Bérubé *et al*.^40^ detected a high degree of genetic differentiation between the two areas; Weir’s *θ*^42^ was estimated at 0.24 and 0.22 for mtCR DNA sequences and microsatellite genotypes, respectively. The high degree of genetic differentiation estimated by Bérubé and co-workers^40,41^, in combination with the very low levels of genetic diversity observed among the Gulf of California fin whales, strongly suggested that this population was reproductively isolated from con-specifics in the eastern North Pacific. However, to date no study has attempted to estimate migration rates between the Gulf of California and the eastern North Pacific or when the Gulf of California population was founded and presumably became isolated. Nigenda-Morales *et al*.^43^ also detected low levels of genetic diversity in the Gulf of California fin whales at the usually highly diverse major histocompatibility complex (MHC) DQB-1 locus. Nigenda-Morales and colleagues^43^ detected only three alleles in a sample of 36 fin whales, a level of MHC diversity comparable to that observed in the severely bottlenecked northern elephant seal, *Mirounga angustirostris*^44^. The low genetic diversity at the mtCR DNA as well as at nuclear microsatellite and MHC loci suggested that effective population size (*N*_*e*_) of the Gulf of California was small, but so far, no actual estimates of *N*_*e*_ have been reported.

The low level of genetic diversity among the Gulf of California fin whales is a function of *N*_*e*_ as well as the duration and degree of isolation. Extended periods at a low *N*_*e*_ increase random genetic drift possibly leading to inbreeding depression^45^ as well as loss of adaptive traits and potentially increasing the overall risk of extinction^46^. The aims of this study were to estimate when the Gulf of California fin whale population was founded, the census (*N*_*c*_) and *N*_*e*_, as well as the degree of connectivity with con-specific populations in the eastern North Pacific. Towards this end, a population genetic assessment of the Gulf of California and eastern North Pacific fin whale specimens was undertaken based upon genotypes at 18 nuclear microsatellite loci in 402 samples and 565 mtCR DNA sequences (including previously published mtCR DNA sequences retrieved from the National Center for Biotechnology Information, NCBI). The estimates suggested that the Gulf of California fin whale population was founded approximately 2.3 thousand years ago (kya), and has since remained at a low *N*_*e*_. The estimates of historic and contemporary connectivity with the eastern North Pacific fin whales were low as well.

## Results

**Figure 1:**
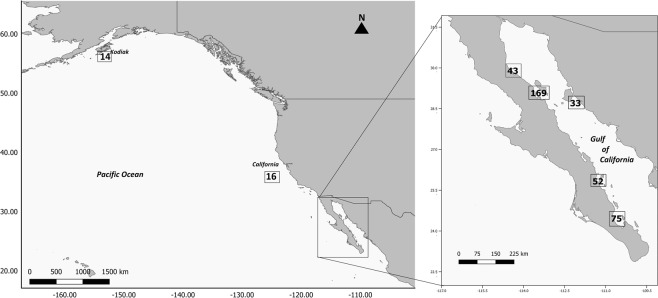
Map of the sampling areas and sample sizes.

A total of 402 tissue samples were collected from individuals off Kodiak Island, off the Californian coast and in the Gulf of California. The sample sizes per year were: Kodiak Island, 14 samples in 2002; coastal California, 16 samples in 1996 and Gulf of California, 372 samples collected in 1993–1995 and 1997–2004 (Table 1 and Fig. 1). The sex, the genotype at 18 nuclear microsatellite loci and the DNA sequence of the 5′-end of the mtCR was determined in all the tissue samples. Two samples from Coastal California and seven from the Gulf of California samples were removed from subsequent data analyses because data were missing at five or more microsatellite genotypes. The assessment of the consistency rate per multi-locus genotype identified a single inconsistent genotype among 470 microsatellite genotypes, yielding an estimate at ~0.2% per genotype. The actual consistency rate in the final dataset was lower since all samples mismatching at four or fewer loci were checked, possibly re-amplified, and corrected if necessary.

**Table 1:** Samples, individuals and genetic diversity of fin whales in three sampling locations.

	Kodiak Island Alaska	Coastal California	Eastern North Pacific	Gulf of California
Tissue samples	14	16	30	372
Nuclear microsatellite genotypes				
*n*	14	11	25	259
*PI*	2.1 × 10^−22^	1.4 × 10^−22^	3.1 × 10^−24^	4.8 × 10^−11^
*N*_*a*_	8.8 ± 3.2	8 ± 2.6	10.8 ± 3.7	7.6 ± 2.4
*H*_*o*_	0.76 ± 0.2	0.82 ± 0.14	0.79 ± 0.15	0.48 ± 0.21
*H*_*e*_	0.81 ± 0.14	0.83 ± 0.08	0.82 ± 0.1	0.49 ± 0.21
* A*_*R*_	7.3 ± 2.36	7.5 ± 2.27	7.5 ± 2.09	3.8 ± 1.32
* F*_*IS*_	0.057	0.015	0.047	0.024
Mitochondrial control region DNA sequences				
*n*	152^&^	160^&^	312^&^	253
*S*	22	19	24	5
*h*_*n*_	25	21	31	4
*θ*	0.014 ± 0.004	0.012 ± 0.004	0.014 ± 0.003	0.003 ± 0.001
*π*	0.008 ± 0.0005	0.006 ± 0.0005	0.007 ± 0.0003	0.0006 ± 0.0001
*h*	0.86 ± 0.02	0.77 ± 0.02	0.82 ± 0.015	0.15 ± 0.03

Notes: *n*, sample size after removing poor quality samples and duplicates; *PI*, probability of identity; *N*_*a*_, the number of alleles; *H*_*o*_, the observed heterozygosity; *H*_*e*_, the expected heterozygosity and *A*_*R*_, allelic richness (based upon 18 individuals); *F*_*IS*_, the inbreeding coefficient; *S*, the number of segregating sites; *h*_*n*_, the number of haplotypes; *θ* per site from *S*; *π*, nucleotide diversity and *h*, haplotype diversity. ^±^Denotes the standard deviation. The Eastern North Pacific is comprised by the Kodiak Island, Alaska and the Coastal California. ^&^Additional mtCR DNA sequences obtained from NCBI.

### Probability of identity and sex ratios

The estimates of the probability of identity (*PI*)^47^ in the three samples were sufficiently low to discern among unrelated individuals. *PI* was estimated at 2.15 × 10^−22^ in the Kodiak Island sample, 1.75 × 10^−22^ in the coastal California sample and 9.08 × 10^−12^ in the Gulf of California sample. Accordingly, the expected numbers of pairs of unrelated individuals with identical genotypes by chance in the sample were estimated at 9.24 × 10^−21^, 8.57 × 10^−21^ and 3.04 × 10^−7^, respectively in the above mentioned samples. Accordingly, all specimens with identical multi-locus microsatellite genotypes, sex and mtCR DNA sequences were inferred as duplicate specimens collected from the same individual, and only data from a single tissue sample were retained in each sample partition during data analysis. No statistically significant deviation from parity was observed for the sex ratio among samples from Kodiak Island, coastal California and the Gulf of California.

The final data set comprised 284 unique individuals totalling 5,092 single-locus genotypes. Six and 14 single-locus microsatellite genotypes were missing among the 25 and 259 unique individuals detected in the eastern North Pacific and Gulf of California sample, respectively. The final mtCR DNA sequence dataset comprised 565 sequences (incl. 284 retrieved from NCBI, see Table S1), each of a length of 280 base pairs. The amounts of data per sampling area, after removal of specimens with insufficient data and duplicate data, are summarised in Table 1.

### Deviations from Hardy-Weinberg genotype proportions and linkage equilibrium

Among the Kodiak Island samples, a single statistically significant deviation (locus EV001) from the expected Hardy-Weinberg genotype proportions was detected, whereas significant deviations were detected at eight loci in the Gulf of California sample after applying a false discovery rate (FDR)^48^ at 0.05 (Table S2). After removing two individuals that were inferred as recent immigrants (see below), the deviations in the Gulf of California sample persisted at five loci (loci EV037, GATA053, GATA098, GGAA520 and GT211, Table S2). Unsurprisingly, the assessment of null alleles suggested the presence of null alleles mostly at the loci that deviated from the expected Hardy-Weinberg genotype frequencies, i.e., Kodiak Island (locus EV001) and the Gulf of California (loci EV037, GATA053, GATA098 and GT575). No significant deviations from linkage disequilibrium were detected after applying FDR.

### Patterns of population genetic diversity and differentiation

Estimates of genetic diversity at nuclear loci and mtCR DNA sequences in sampling areas outside the Gulf of California yielded values approximately twice the levels observed in the Gulf of California (Table 1). The level of genetic differentiation was high for the mtCR DNA sequences and nuclear loci in comparisons involving the Gulf of California (Table 2). The lowest level of population genetic differentiation was detected between the Kodiak Island and the coastal California samples (*ϕ*_*ST*_ = 0.027 for the mtCR DNA sequences and *F*_*ST*_ = 0.016, 95% confidence interval (CI): 0.004–0.03 for the microsatellite genotypes; Table 2). Since the degree of population genetic differentiation between the two eastern North Pacific samples was one order of magnitude lower than comparisons including the Gulf of California, the two eastern North Pacific samples were combined into a single “eastern North Pacific” sample during subsequent data analyses. The eastern North Pacific samples were included in the analysis specifically as a means to gain insights into the history and isolation of the Gulf of California population. The aim of this study was not to make inferences concerning potential connectivity and divergence times among eastern North Pacific sample areas *per se*, given the modest sample sizes available from the eastern North Pacific region.

**Table 2:** Population differentiation estimates.

	Kodiak Island, Alaska	Coastal California	Gulf of California
Kodiak Island, Alaska		0.027**	0.25***
Coastal California	0.016* [0.004–0.03]		0.19***
Gulf of California	0.25*** [0.17–0.34]	0.21*** [0.13–0.29]	

Notes: Above the diagonal; *ϕ*_*ST*_ estimated from the mtCR DNA sequences. Below the diagonal; *F*_*ST*_, estimated from the microsatellite genotypes along with the 95% confidence interval in brackets. *P ≤ 0.05; **P ≤ 0.01 and ***P ≤ 0.001.

### Contemporary estimates of *N*_*e*_*m*, *N*_*e*_ and *N*_*c*_

Contemporary immigration rates were estimated using the approach implemented in BAYESASS. The Markov chain Monte Carlo (MCMC) mixing parameters for the migration rates was at 0.07, the allele frequencies at 0.2 and inbreeding coefficients at 0.15 (for convergence, Fig. S1). The number of immigrants per generation (*N*_*e*_*m*) from the eastern North Pacific into the Gulf of California (Table 3 and Fig. S2) was estimated at ~1.4 (95% High Posterior Density (HPD): ~0.12–5.39, effective sample size (ESS): 2,444) employing an estimate of *N*_*e*_ at 360 (see below). Excluding loci with possible null alleles from the assessment did not alter the estimates (Table S3). A single first (migration ancestry = 1) and a single second-generation immigrant (migration ancestry = 0.99) were identified among the Gulf of California samples.

**Table 3:** Estimates of effective population size, divergence times and immigration rates.

	*N*_e(ENP)_*	*N*_e(GC)_*	*N*_e(A)_*	*T* ^&^	*m*_( *GC*→ *ENP*)_	*m*_( *ENP*→ *GC*)_	*N*_e(GC)_ *m*_( *ENP*→ *GC*)_
	**MSVAR**				**BAYESASS**		
msat	NA	253	12,500	19,000	0.020	0.004	1.4
		(126–516)	(5,900–26,800)	(9,200–38,700)	(0–0.06)	(0.0005–0.009)	(0.12–5.39)
	**IMA2P**						
mtCR	28,300	330	21,700	1,370	0.0008	0.0002	0.89
	(16,400–47,900)	(0–2,300)	(0–125,300)	(0–2,700,000)	(0.0002–0.002)	(0–0.0047)	(0.04–3.13)
mtCR & msat	6,500	40	7,400	2,300	0.0003	0.002	—
	(2,300–60,800)	(0–700)	(4,900–12,700)	(0–62,500)	(0–0.002)	(0–0.015)	—

Notes: *ENP, GC and A, denotes the eastern North Pacific, Gulf of California and ancestral population, respectively. ^&^Estimated timing of the event in years. *N*_*e*_, denotes the effective population size and *m* the immigration rate. mtCR, denotes the mitochondrial control region DNA sequences and msat, denotes microsatellite genotypes. The contemporary immigration rates from BAYESASS represent the fraction of individuals in the eastern North Pacific that are immigrants from the Gulf of California per generation (*m*_(*GC→NP*)_) and the fraction of individuals in the Gulf of California that are immigrants from the eastern North Pacific (*m*_(*NP→GC*)_). In order to facilitate a comparison with the estimates of the long-term immigration rates per generation obtained with IMA2P (Tables S6 and S7) which are scaled to the mutation rate, IMA2P estimates were transformed to *m*. The mutation rate for the transformation of the mtCR data was 5.2 × 10^−8^ per site per year, considering 280 base pairs and a generation time of 25.9 years. The mutation rate for the transformation of the combined dataset was 5 × 10^−4^ per generation, which is the geometric mean of the mtCR and msat mutation rate. The contemporary number of immigrants per generation from the North Pacific to the Gulf of California (*N*_*e*(*GC*)_*m*_(*NP*→*GC*)_) was estimated using the estimates obtained with BAYESASS and the estimate of *N*_*e*_ obtained with NEESTIMATOR. The long-term *N*_*e*_*m* was estimated using IMA2P. *N*_*e*(*GC*)_*m*_(*NP*→*GC*)_ for the combined data (mtCR and msat) was not estimated (see Fig. S8) but the mutation rate scaled by the mutation rate was (*m*, Fig. S9).

The linkage disequilibrium-based estimate of *N*_*e*_ was 360 [95% CI: 230–665] in the Gulf of California sample. *N*_*c*_ in the Gulf of California was estimated from 259 unique individuals, among which one, five and 40 individuals were sampled in four, three, or two different years, respectively (Table S4). The remaining individuals were sampled only once. The above re-capture histories yielded an estimate of *N*_*c*_ at 730 [95% CI: 530–930] individuals after model averaging (Table S5). The *N*_*c*_ estimate refers to the total number of individuals that existed during the study period.

### Effective population sizes and divergence time

A possible change in the effective population size in the Gulf of California fin whales was estimated using MSVAR^49^. The MSVAR estimate (microsatellite genotypes only) obtained from the Gulf of California samples yielded an estimate of an ancestral *N*_*e*_ at 12,000 (ESS: 5,220), which was reduced to an *N*_*e*_ at ~250 (ESS: 3,007) approximately 19 kya [95% HPD: 9.22–38.7 kya, ESS: 3,075] (Table 3; Figs S3 and S4). The population divergence time, long-term *N*_*e*_, and immigration rates were estimated using the parallelised version of the isolation-with-migration model^50^ implemented in IMA2P^51^. The IMA2P parameter estimates differed among the data employed in the analysis but were consistent with the MSVAR results in terms of a low *N*_*e*_ for the Gulf of California. The IMA2P assessment based solely on mtCR DNA sequences yielded a *N*_*e*_ for the Gulf of California at ~330 [95% HPD: 0–2,300; ESS: 75,588]. The IMA2P assessment based upon both mtCR DNA sequences and microsatellite genotypes resulted in an estimate of *N*_*e*_ at ~40 [95% HPD: 0–700; ESS: 1,202]. The estimate of the divergence time between the Gulf of California and the eastern North Pacific was estimated at ~1.3 kya [95% HPD: 0–2,700 kya; ESS: 559,148] when employing only mtCR DNA sequences and ~2.3 kya [95% HPD: 0–62.5 kya; ESS: 4,615] for the combined data (Tables 3, S6 and S7; Figs S5 and S6). *N*_*e*_*m* following population divergence was estimated at 0.89 [95% HPD: 0.03–3.13; ESS: 49,663] for the mtCR DNA sequences (Tables 3 and S6; Fig. S7). However, obtaining an estimate of *N*_*e*_*m* from the combined data was not feasible (Tables 3 and S7; Figs S8 and S9).

Because the 95% HPD interval of the IMA2P estimate of the population divergence time included zero, the relative probabilities of a recent and an older population divergence time were estimated from the mtCR DNA sequences and microsatellite genotypes using the approximate Bayesian computation (ABC)^52^ approach implemented in the software DIYABC^53^. The scenario with an older population divergence time (>1 kya) was preferred with a posterior probability at 0.89 [95% CI: 0.88–0.9] using the logistic approach (Table S8, Figs S10 and S11). The average type I and II error rates were estimated at 6.6% and 2.8%, respectively.

### MtCR DNA sequence haplotype network

**Figure 2:**
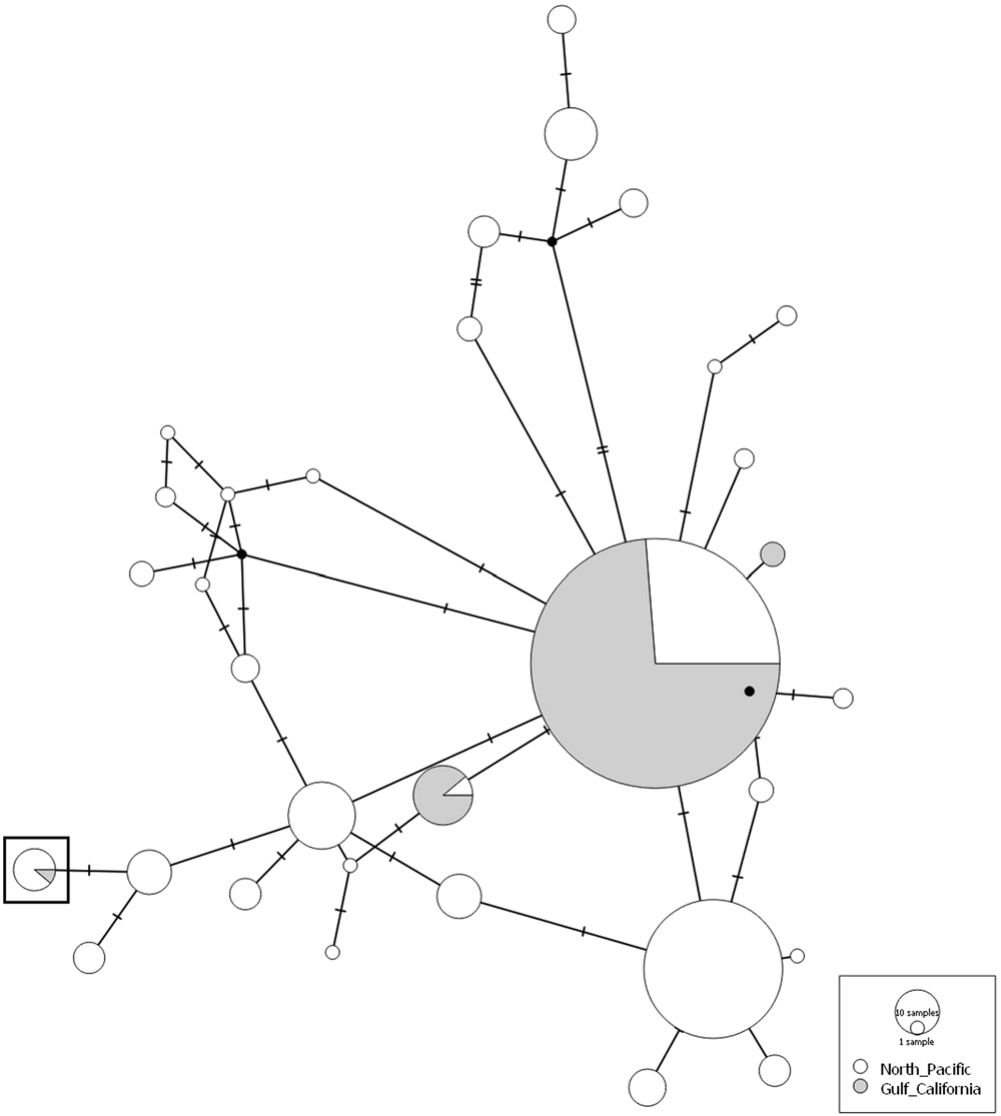
Media Joining network inferred from fin whale mtCR DNA sequence haplotypes. The haplotype of the individual sampled in the Gulf of California inferred to be a first-generation immigrant is marked with a square.

Only four mtCR DNA sequence haplotypes were detected in the Gulf of California sample, of which three were also detected in the eastern North Pacific sample (Fig. 2). One of the mtCR DNA sequence haplotypes that was detected both in the eastern North Pacific and the Gulf of California samples was identified in a single Gulf of California individual, an individual inferred to be a first-generation immigrant (haplotype marked with a square in Fig. 2). A single mtCR DNA sequence haplotype accounted for ~92% (238 of 259) of the Gulf of California individuals.

## Discussion

The analyses in this study revealed that the Gulf of California fin whale population was founded approximately 2.3 kya and subject to very limited gene flow from the neighbouring populations in the eastern North Pacific. The contemporary *N*_*e*_ and *N*_*c*_ estimates for the Gulf of California population (~360 and 730, respectively) were low as was the IMAP2 estimate of the “long-term” *N*_*e*_ (~40) suggesting that this fin whale population has remained small since it was founded, which in turn explains the very low levels of contemporary genetic diversity in this population. The contemporary, highly productive, oceanographic conditions in the Gulf of California were established ~2.8 kya^54,55^, which correspond remarkably well with the IMAP2 estimate of the divergence time between the Gulf of California and eastern North Pacific fin whales. The primary productivity in the Upper Gulf of California can reach very high levels (364 mgC m^−3^ D^−1^)^56^ seemingly resulting in conditions that can sustain a resident population.

Our results supported the notion that the establishment of the Gulf of California fin whale population was not a recent phenomenon. The DIYABC-based assessment yielded strong support for a divergence time between the Gulf of California population and the eastern North Pacific more than 40 generations ago (>1 kya). The IMA2P divergence time estimate agreed with the DIYABC-based assessment, suggesting a reduction from an ancestral *N*_*e*_ at ~7,000 to ~40 approximately 2.3 kya. The MSVAR assessment also suggested a similar reduction, although the timing of the change in *N*_*e*_ inferred from the MSVAR assessment was much older than the IMA2P estimate, but included in the 95% HPD of the IMA2P estimate. The IMAP2 estimates accounted for migration, which was not the case for the estimates obtained using DIYABC or MSVAR. The MSVAR estimate could also reflect another, older change in *N*_*e*_. Despite the quantitative differences, the qualitative conclusion among these slightly different analytical approaches was similar; i.e., the Gulf of California fin whale population was founded more than one kya, and most likely around 2.3 kya.

The estimates of migration rates between the Gulf of California and the eastern North Pacific were well below the values viewed as evidence for evolutionary independence^57–59^. The contemporary and “long-term” *N*_*e*_*m* were estimated at ~1.4 and 0.9 respectively. All estimates of migration rates (and effective population sizes) are sensitive to ghost populations (current unsampled, and even extinct populations)^59,60^. However, the distinct and very low levels of genetic diversity in the Gulf of California fin whales compared to the adjacent eastern North Pacific fin whale populations (see Archer *et al*.^61^) suggest that immigration from these adjacent populations has been minimal, and hence likely not of major concern in this case. Moreover, the Gulf of California is a semi-enclosed sea, hence immigration from other populations besides the eastern North Pacific was unlikely.

The estimated contemporary immigration rates were roughly consistent with the identification of only two individuals with an immigrant ancestry among the 259 individuals sampled in the Gulf of California during the 12-year sampling period. One of the two individuals with an immigrant ancestry, was a male inferred as a first-generation immigrant, whereas the second “immigrant” individual was inferred as a F1, i.e., the offspring of an immigrant individual. The latter individual was previously reported by Bérubé and colleagues^40^. Overall the results obtained during this study were in agreement with the inferences drawn in previous genetic^40,41^ and acoustic assessments^38,39,60^, as well as the restricted range observed in satellite-tagged fin whales in the Gulf of California^34,37^ and the scarcity of fin whales sightings at the southern tip (south of 24°N) of the Baja California Peninsula^31,33–35^.

The degree of population genetic differentiation between the Gulf of California and the eastern North Pacific was relatively high (*F*_*ST*_ ~ 0.2 for microsatellite genotypes and *Φ*_*ST*_ ~ 0.2 for mtCR DNA sequence data), compared to levels observed among other con-specific populations in other baleen whale species in the same oceanic basin^40^. Fin whales in the Mediterranean Sea also appeared to be (at least in part) a resident population^41^. The degree of differentiation using mtCR DNA sequences between the Mediterranean Sea fin whales and their eastern North Atlantic con-specifics was slightly lower (ranging from 0.09 to 0.15)^41^ compared to the Gulf of California and eastern North Pacific in this study. *N*_*e*_*m* between the North Atlantic and the Mediterranean Sea fin whales was estimated at two (female) migrants per generation^61^. However, the degree of genetic differentiation between the North Atlantic and the Mediterranean fin whale populations at nuclear microsatellite genotypes was much lower (*F*_*ST*_ ~ 0.007), perhaps due to occasional male-mediated gene flow^61,62^.

The results presented here suggested that the low genetic diversity in the Gulf of California fin whales was a product of a low, long-term *N*_*e*_ and a low *N*_*e*_*m*. The low genetic diversity among the Gulf of California fin whales has been noted earlier^40,41,43^. Other studies have reported low estimates of *N*_*e*_ in specific baleen whale populations, such as eastern Canada and Sea of Okhotsk bowheads^63^, as well as among right whales in the South Atlantic and Indo-Pacific ocean basins^64^. However, the levels of genetic diversity reported for these populations were much higher (*H*_*o*_ = 0.71–0.83) than the genetic diversity observed among Gulf of California fin whales. An illustration of the very low genetic diversity among the Gulf of California fin whales was the observation that a single mtCR DNA sequence haplotype accounted for ~92% of all the sampled individuals. The mtCR DNA sequence haplotype (*h*) and nucleotide diversity (*π*) was estimated at 0.14 and 0.0006, respectively, which were among the lowest values reported for cetaceans^40,41,65–69^. For instance, reported estimates of mtCR DNA sequence diversity in the highly endangered western North Atlantic right whale (*Eubalaena glacialis*) were substantially higher (ℎ = 0.69 and 𝜋 = 0.006)^66^.

The very low level of genetic diversity among the Gulf of California fin whales is of potential concern in terms of loss of adaptive traits and a possible increase in genetic load^5,46,70–72^. A few recent genomic-based studies have detected an increase in genetic load at a low *N*_*e*_. Pedersen *et al*.^73^ identified elevated levels of detrimental alleles among Greenlandic Inuit (*Homo sapiens*), which underwent a bottleneck for 15–20 kya at a *N*_*e*_ at ~1,000. Rogers and Slatkin^74^ identified an apparent increase in the number of detrimental mutations in a small isolated population of woolly mammoth (*Mammuthus primigenius*) on Wrangel Island, which underwent a bottleneck of a similar extent and duration to that inferred for the Greenlandic Inuit. Both examples confirmed the expected increase in detrimental traits following a protracted population bottleneck and hence a reduction in population fitness. In both examples, the negative effects were observed in populations with a *N*_*e*_ larger than the estimated in the Gulf of California fin whale population. However, the duration of the bottleneck in the two mentioned examples was much longer.

On the other hand, there have also been examples of mammal populations that persisted for thousands of years despite low levels of genetic diversity, such as the endangered Iberian lynx (*Lynx pardinus*), which appeared to have persisted at a *N*_*e*_ at 200–300 for 8.1–50 kya^75^. Among marine mammals very low levels of mtCR DNA sequence diversity has been reported in the narwhal (*Monodon monoceros*)^69^ with no apparent adverse effects and a large contemporary populations size (~24,000 individuals^76^). In contrast to the narwhal, *N*_*c*_ of the Gulf of California fin whale population was estimated at only 730 individuals. The genetic tagging^8^ estimate of *N*_*c*_ for the Gulf of California fin whale population was concordant with previous *N*_*c*_ estimates based upon line transect surveys (ranging from 297 to 820)^34^ and a single mark-recapture estimate (600) based upon individual identification from natural markings^77^.

The results presented here implies that Gulf of California fin whales could be subject to elevated rates of genetic load and loss of adaptive variation, although the population appeared to have persisted at a low *N*_*e*_ for ~2.3 kya. Assessing the contribution of low genetic variation upon the population extinction rate in a species, such as fin whales is non-trivial. Unlike most other baleen whale populations, the Gulf of California fin whales were not subjected to commercial exploitation. However, this small population is currently exposed to a multitude of recent anthropogenic pressures, including bycatch (Urban and Brownell, unpublished data), entanglement in fishing gear, ship strikes, possible disturbance by whale-watching boats, anthropogenic sound^78,79^ and habitat degradation due to chemical pollution, microplastics^80^ and urban development^35^.

Such anthropogenic effects on mortality and reproductive rates may add to the genetic effects stemming from a low *N*_*e*_ through several thousand years. The low *N*_*e*_ and *N*_*c*_ of the Gulf of California fin whale population, coupled with the very low connectivity with the eastern North Pacific fin whales documented here, indicates that a cautionary management scheme should be considered in order to avoid this population joining the other small baleen whale populations currently declining from anthropogenic threats^81–83^, especially bycatch^84^, entanglements and ship strikes.

## Methods

### Sample collection

Tissue samples were collected as skin biopsies^85^ and sloughed skin^86^ from free-ranging fin whales, or during necropsies of beached fin whales. Tissue samples were collected by Universidad Autónoma de Baja California Sur with the approval and permits issued by the Mexican Wildlife Agency (Dirección General de Vida Silvestre, Subsecretaría de Gestión para la Protección Ambiental, Secretaría del Medio Ambiente y Recursos Naturales). Samples from US waters were collected by the Southwest Fisheries Science Center and by the National Marine Mammal Lab in accordance with national guidelines and regulations. The research permits also included the necessary ethical approval in terms of sample collection. All sample collection was undertaken in accordance with relevant national guidelines and regulations. Tissue samples were preserved in a saturated sodium chloride solution with 20% dimethyl sulfoxide^87^ and stored at −20 degrees Celsius (°C).

### Laboratory analyses

Genomic DNA was extracted by either standard phenol-chloroform extractions^88^ or using QIAGEN DNEasy^TM^ extraction columns for animal tissue, following the manufacturer’s instruction (QIAGEN Inc.). The quality of the extracted DNA was visually checked by electrophoresis through a 0.7% agarose gel, and quantified with a Qubit^TM^ following the manufacturer’s instructions (Thermo Fisher Scientific Inc.). DNA extractions were normalized to a concentration of 20 ng DNA/μL. Sex was determined by the differential amplification of the zinc finger coding regions, ZFX and ZFY^89^ as well as by co-amplification of a SRY-specific region with one or more autosomal microsatellite loci (Bérubé, *in prep*).

The genotypes were determined at 18 microsatellite loci: GATA028^90^, GATA053^90^, GATA098^90^, GATA417^90^, GGAA520^90^, TAA023^90^, GT011^41^, EV001^91^, EV037^91^, EV094^91^, GT023^92^, GT575^92^, GT195^92^, GT211^92^, GT271^92^ GATA25072, GATA43950 and GATA6063862 (last three loci, Bérubé, *in prep*). Negative and positive controls were included with each set of polymerase chain reaction^93^ (PCR) amplifications. Individual PCR amplifications were carried out as described by Palsbøll, *et al*.^90^ and Bérubé, *et al*.^41,92^. PCR fragments were separated by electrophoresis on an ABI 3730 DNA Analyzer™ (Applied Biosystems Inc.) with GeneScan^TM^-500 ROX (Applied Biosystems Inc.) as size standard. The length of each PCR product was determined with GENEMAPPER™ (ver. 4.1, Applied Biosystems Inc.). The consistency rate per multi-locus genotype was estimated by re-amplifying ten loci (GATA028, GATA053, GATA098, GATA417, GGAA520, TAA023, GT011, GT575, GT195, and GT211) in 47 randomly selected DNA extractions.

The first 285 base pairs of the 5′-end of the mtCR were amplified using the oligo-nucleotides MT4F^94^ and BP16071R^95^. The PCR amplifications were performed in 20 μL reaction volumes with 0.2 μM of each dNTP, 67 mM Tris-HCl (pH 8.8), 2 mM MgCl_2_, 17 mM NH_3_SO_4_, 10 mM *β*-mercaptoethanol, 0.1 μM of each oligo-nucleotide, 0.4 units of *Taq* DNA polymerase (New England BioLabs® Inc) and 10–20 ng of DNA extraction. The thermo-cycling profile was 2 min at 94 °C, followed by 25 cycles each with 15 seconds at 94 °C, 30 seconds at 54 °C and 120 seconds at 72 °C. Unincorporated nucleotides and excess oligo-nucleotides were enzymatically removed using *shrimp alkaline phosphatase* and *exonuclease* I as described by Werle, *et al*.^96^. Cycle-sequencing was conducted according to the manufacturer’s instructions using Big Dye™ (ver. 3.1, Life Technologies Inc.) with the oligo-nucleotides employed in the initial PCR amplification. The cycle-sequencing products were precipitated with 96% ethanol^88^ and re-suspended in 10 μL deionized formamide (Calbiochem^®^ Inc.). The order of cycle-sequencing products was resolved by electrophoresis on an ABI 3730 DNA Analyzer™ (Applied Biosystems Inc.). The reverse strand was sequenced in DNA extractions with unique mtCR DNA sequence haplotypes. Additional mtCR DNA sequences from Bérubé, *et al*.^41^ and Archer, *et al*.^97^ were retrieved from NCBI (Table S1).

### Data analysis

#### Probability of identity and sex ratios

*PI*^47^ was estimated using CERVUS (ver. 3.0.7)^98^ assuming that all individuals were unrelated, in order to determine the probability that two unrelated individuals had identical multi-locus genotypes.

The expected number of pairs of unrelated individuals with identical genotypes by chance in the sample was estimated by multiplying the number of pairwise comparisons by *PI*. The *PI* was also used to estimate the number of loci needed to identify an individual^99^, and this threshold was used to check the samples with few mismatches. The probability of an observed deviation from parity of the sex ratio was estimated as the fraction of 2,000 random permutations of the data in which *χ*^2^ was equal to or larger than the observed value of *χ*^2^ by means of a *χ*^2^ test in R (ver. 3.0.1)^100^.

#### Deviations from Hardy-Weinberg genotype proportions and linkage equilibrium

Departures from Hardy-Weinberg equilibrium and linkage disequilibrium were estimated using ARLEQUIN (ver. 3.5.2.2)^101^. Departures from Hardy-Weinberg equilibrium were assessed for each microsatellite locus in each sample from 10,000 dememorization MCMC steps followed by 100,000 steps. The probability of linkage disequilibrium between all pairs of loci per population were estimated from 10,000 permutations. The significance of the departures from Hardy-Weinberg equilibrium and linkage equilibrium was corrected using FDR^48^ at 0.05. The possible presence of null alleles, large allele dropout and scoring errors due to stuttering was assessed using MICRO-CHECKER (ver. 2.2.3)^102^.

#### Patterns of population genetic diversity and differentiation

The number of alleles (*N*_*a*_) as well as the observed (*H*_*o*_) and expected heterozygosity (*H*_*e*_) were estimated using ARLEQUIN. Allelic richness (*A*_*R*_) was estimated with the package ADZE (ver.1.0)^103^ for all 18 microsatellite loci in sample sizes of 18 (the smallest sample size). The inbreeding coefficient (*F*_*IS*_)^104^ was estimated as implemented in FSTAT (ver. 2.9.3.2)^105^. The mtCR DNA sequences were aligned using the Clustal W algorithm^106^ implemented in MEGA (ver. 6.06)^107^ with the default settings. DNASP (ver. 5.10.1)^108^ was employed to estimate the number of segregating sites (*S*) and haplotypes (*h*_*n*_) as well as *π*, *h* and *θ* (per site) from *S*^109^.

The degree of population genetic differentiation was estimated as *ϕ*_*ST*_ from the mtCR DNA sequences using ARLEQUIN^101^ from the number of pairwise differences. In case of the microsatellite genotypes, the population genetic differentiation was estimated as *F*_*ST*_ using FSTAT (ver.2.9.3.2)^105^. The 95% CI was estimated from 15,000 permutated datasets by bootstrapping loci.

### Contemporary estimates of *N*_*e*_*m*, *N*_*e*_ and *N*_*c*_

“Contemporary” immigration rates were estimated from the microsatellite genotypes employing the Bayesian approach implemented in the software BAYESASS (ver. 3.0.4)^110^. Preliminary estimations were performed to adjust the MCMC mixing parameters of migration rates, allele frequencies, and inbreeding coefficients to acceptance rates at 20–60%. A total of 11 independent MCMCs were performed, each with different random starting seeds and a burn-in of 1,000,000 steps followed by 10,000,000 steps sampling every 1,000th step. The final parameter estimates were inferred from the estimation with the lowest Bayesian deviance using the R script authored by Meirmans^111^.

Contemporary *N*_*e*_ was estimated from the degree of linkage disequilibrium among the microsatellite loci using the bias-corrected approach by Waples and Do^112^ as implemented in NEESTIMATOR (ver. 2.1)^113^. The estimation was conducted excluding the first-generation immigrant detected during the BAYESASS assessment because the linkage disequilibrium approach in NEESTIMATOR assumes that all genotypes originate from a single panmictic population. The second-generation migrant was included in the dataset because the individual was part of the Gulf of California population. The critical minimum allele frequency was set to 0.02. The 95% CI of each point estimate was estimated by jack-knifing among samples^114^.

The census population size (*N*_*c*_) was estimated using the POPAN derivation of the Jolly-Seber model as implemented in MARK (ver. 6.1)^115^ via the *RMark* package^116^. Each year was employed as a single capture event (Table S4). The Akaike’s information criterion with a correction for small sample size was employed to assess the model support. Model averaging was performed in MARK^115^.

### Population sizes and divergence time

A possible change in effective population size in the Gulf of California sample was estimated using MSVAR (ver. 1.3)^49^. Loci with “micro-variant” alleles^117^ causing imperfect repeats were excluded: GATA053, GATA417, GGAA520 and GATA25072). Two duplicate runs were conducted, each with different random seeds using the exponential population size change model. Starting values of each duplicate run were initiated from the upper and lower range of the prior distribution of each parameter (current population size, *N*_0_ = 300, ancestral population size, *N*_1_ = 10,000, mutation rate, *μ* = 0.0005 and time of population size change, *T*_*α*_ = 16,000). Each MCMC comprised of 10,000,000 steps with sampling at each 1,000^th^ step. The first 5,000 of the 10,000 recorded steps were discarded before assessing convergence and parameter estimation.

The population divergence time, long-term *N*_*e*_, and immigration rates were estimated using the parallelised version of the isolation-with-migration model^50^ implemented in IMA2P^51^. Two different datasets were analysed; a dataset comprised solely of mtCR DNA sequences and another dataset including both mtCR DNA sequences and microsatellite genotypes. Microsatellite loci with “micro-variant” alleles were excluded from this estimation as well. The Hasegawa-Kishino-Yano, HKY, substitution model^118^ was employed with a mutation rate at 5.2 × 10^−8^ per site per year^119^ for the mtCR DNA sequences. The mutation rate per locus was based upon 280 base pairs. A stepwise mutation model was employed for the microsatellite genotypes using a mutation rate at 7 × 10^−4^ per generation (estimated in the MSVAR analysis). A generation time at 25.9 years^120^ was used for converting the generational mutation rate into an annual mutation rate. Preliminary estimations were conducted to identify the optimal parameter prior ranges. The final estimates for the mtCR DNA sequences were based upon three replicate estimations: each with ten MCMCs consisting of 400,000 steps, of which the first 200,000 were discarded, and sampling each 100th step. In addition, one run was started using the Markov chain state generated in a previous run.

For the dataset including both mtCR DNA sequences and microsatellite genotypes a random sample of 30 individuals from the Gulf of California was employed in the analysis, in order to avoid effects stemming from unequal sample sizes between the eastern North Pacific and the Gulf of California. The estimations for the combined data were based upon four replicates: each with ten MCMCs consisting of 250,000 steps, of which the first 150,000 were discarded, and a sampling interval at every 100th step. In addition, two runs were started using the Markov chain state generated in previous runs. Reported estimates were those obtained from the run with the highest ESS.

Because the 95% HPD interval of the IMA2P estimate of the population divergence time included zero, DIYABC (ver. 2.1)^53^ was used to test between a recent (scenario 1: 0–20 generations ago) versus an older (scenario 2: 40–900 generations ago) population divergence time. The point estimate of the population divergence time given by IMA2P analysis using only mtCR DNA sequences was used as the starting point for the divergence time distribution of scenario 2 (40 generations ago, which is ~1,000 years ago). The divergence time distribution of scenario 1 starts at zero and ends at 20 generations in order to not overlap with scenario 2. DIYABC assessments were based upon two million simulated datasets for each scenario using mtCR DNA sequences and microsatellite genotypes. Uniform prior distributions were used for the demographic parameters: North Pacific effective population size, *N*_1_ [10,000–80,000]; Gulf of California effective population size, *N*_2_ [1–1,000], scenario 1 divergence time, *T*_1_ [0–20] and scenario 2 divergence time, *T*_2_ [40–900]. A HKY mutation model was used for the mtCR DNA sequences with a uniform distribution from 1 × 10^−7^ to 1 × 10^−5^ and 96% of invariable sites. Default values were used for the other mutation model parameters. The posterior probability of each scenario was estimated from a logistic regression performed on one percent of the simulated datasets closest to the observed values after a linear discriminant analysis^121^. The confidence in the scenario choice was evaluated by estimating the type-I and type-II error rates for 1,000 simulated datasets in a linear discriminant analysis. The model checking (Table S8) was performed as implemented in DIYABC.

MCMC-based estimations were deemed to have converged when estimates were consistent among independent replicate MCMC runs (minimum three, except MSVAR) and when effective sample sizes (ESS) were above 200. The ESS, trace plots, and Gelman-Rubin diagnostic were estimated for MSVAR and BAYESASS using the R package *coda*^122^.

### MtCR DNA sequence haplotype network

A median-joining mtCR DNA sequence haplotype network^123^ was inferred using POPART (ver. 1.7)^124^ with *ε* set at zero.

## Supplementary information

Supplementary_material

## Data Availability

The datasets generated during the current study are available at Datadryad.org under accession: 10.5061/dryad.s07g211.
