# Supplementary material for: Long-term isolation at a low effective population size greatly reduced genetic diversity in Gulf of California fin whales
Source: Sci Rep. 2019 Aug 27;9:12391. doi: 10.1038/s41598-019-48700-5 (PMC6712047; doi:10.1038/s41598-019-48700-5)
Supplement: Supplementary file 1 — Supplementary_material [file 41598_2019_48700_MOESM1_ESM.pdf]

Long-term isolation at a low effective population size greatly reduced genetic diversity in Gulf of California fin whales

Vania E. Rivera-León[1*], Jorge Urbán[2], Sally Mizroch[3], Robert L. Brownell Jr.[4], Tom Oosting[1], Wensi Hao[1], Per J. Palsbøll[1,5*$] and Martine Bérubé[1,5*$]

(1) *Marine Evolution and Conservation, Groningen Institute of Evolutionary Life Sciences, University of Groningen, Nijenborgh 7, 9747 AG, Groningen, The Netherlands*

(2) *Departamento de Ciencias Marinas y Costeras, Universidad Autónoma de Baja California Sur, Km 5.5 Carretera al Sur, 23081, La Paz, Baja California Sur, México*

(3) *mBlue Sea Research PO Box 15805 Seattle, WA 98115, United States of America*

(4) *Southwest Fisheries Science Center, NOAA Fisheries, 34500 Highway 1, Monterey, CA 93940, United States of America*

(5) *Centre for Coastal Studies, 5 Holway Avenue, Provincetown, Massachusetts 02657, United States of America*

[*]*Corresponding authors: v.e.rivera.leon@rug.nl/p.j.palsboll@rug.nl/m.berube@rug.nl; Telephone: +31 50 363 9882/+31 50 36 32393*

[$]*Shared senior authorship*

SUPPLEMENTARY MATERIAL

Tables

Table S1. NCBI mitochondrial control region (mtCR) DNA sequence accession numbers

Reference	Accession number
Bérubé et al. (1998)	AF119956.1 to AF119977.1<br>AF120004.1 to AF120006.1
Archer et al. (2013)	KC572711, KC572715, KC572717 - KC572725, KC572727 - KC572730, KC572734 - KC572744, KC572746 - KC572777, KC572779 - KC572781, KC572783 - KC572786, KC572807 - KC572824, KC572855, KC582023, KC582041 - KC582044, KC582047 - KC582059, KC582063 - KC582150, KC582152 - KC582166, KC582193 - KC582215, KC582231 - KC582234 and KC582242 - KC582296

Table S2. Probability of single-locus deviations from the expected Hardy-Weinberg genotype proportions

Locus	Kodiak Island (n = 14)	Coast of California (n=11)	Gulf of California[1] (n=259)	Gulf of California[2] (n=257)
EV001	0 (0.25)*	0.51	0.98	0.98
EV037	0.2	0.94	0 (0.05)*	0.0001(0.05)*
EV094	0.3	0.74	0.79	0.61
GATA028	0.94	0.60	0.85	0.84
GATA053	0.10	0.3	0 (0.12)*	0(0.11)*
GATA098	0.42	0.17	0.004 (0.06)*	0.009(0.06)*
GATA25072	0.42	0.81	0.88	0.92
GATA417	0.94	0.74	0.56	0.66
GATA43950	0.72	0.36	0.35	0.40
GATA6063862	0.5	1	0.38	0.31
GGAA520	0.08	0.84	0.004*	0.004*
GT011	0.55	0.43	0.32109	0.44
GT023	0.34	0.92	0.005*	0.34
GT195	0.32	0.90	0.01*	0.04
GT211	0.05	0.78	0.003*	0.01*
GT271	0.63	0.31	0.08	0.1
GT575	0.48	0.10	0 (0.05)*	0.04(0.05)
TAA023	0.72	0.94	0.25	0.2
FDR	0.003	0.003	0.022	0.014

Notes: P-values marked with an asterisk were < 0.05 after applying a FDR at 0.05. The frequencies of inferred, possible, null-alleles are denoted in parentheses. [1]All unique multi-locus genotypes. [2]Excluding multi-locus genotypes inferred as immigrants.

Table S3. BAYESASS-based estimates of contemporary immigration rates

$m_{(ENP\rightarrow ENP)}$	$m_{(GC\rightarrow GC)}$	$m_{(ENP\rightarrow GC)}$	$m_{(GC\rightarrow ENP)}$
0.97 ± 0.019	0.996 ± 0.002	0.0035 ± 0.002	0.02 ± 0.019
[0.936 - 0.999]	[0.991 - 0.999]	[0.0002 – 0.009]	[0.001 - 0.06]

Notes: The values denote the median of the posterior distribution ± one standard deviation. Square brackets denote the 95% HPD interval. The numbers represent the fraction of immigrant individuals per generation and → denotes the direction of immigration. The estimations were carried out without the loci presenting null alleles, which are EV037, GATA053, GATA098 and GT575.

Table S4. Unique fin whale individuals and recaptured individuals sampled from 1993 to 2004.

Year	n	m	M
1993	17	0	0
1994	44	4	17
1995	16	1	57
1996	0	0	72
1997	3	1	72
1998	30	2	74
1999	17	2	102
2000	31	12	117
2001	11	5	136
2002	2	0	142
2003	11	3	144
2004	134	27	152

Notes: n, unique individuals per year; m, number of recaptured individuals (previously marked) in the sample; M, number of marked individuals in the population prior to the sample.

Table S5. Estimates of N_c in the Gulf of California

Model	Weight	N_c	SE	AICc	Delta AICc
Φ(~Time), p(~time), β(~time), N(~1)	0.43	760.41	96.46	478.21	17.88
Φ(~1), p(~time), β(~time), N(~1)	0.26	702.2	84.22	476.85	16.52
Φ(~1), p(~time), β(~1), N(~1)	0.1	684.44	77.07	460.33	0
Φ(~1), p(~time), β(~Time), N(~1)	0.09	729.95	85.75	460.44	0.11
Φ(~Time), p(~time), β(~1), N(~1)	0.04	708.33	87.42	461.92	1.59
Φ(~Time), p(~time), β(~Time), N(~1)	0.04	802.44	153.44	462.26	1.93
Φ(~time), p(~time), β(~1), N(~1)	0.02	622.88	92.22	480.18	19.85
Φ(~time), p(~time), β(~Time), N(~1)	0.01	768.07	269.23	481.66	21.33
Φ(~time), p(~time), β(~time), N(~1)	0.01	693.33	191.31	499.40	39.08

Notes: Φ, survival; p, probability of capture; β, probability of entry into the population; (~1) the parameter is constant over time; (~Time) the parameter has a linear temporal trend; (~time) the parameter has a variable temporal trend. All the models were used to obtain the model averaged N_c (N) estimated at 731.47 [95% CI: 529.45 – 933.49].

Table S6. IMA2P estimates using only mtCR DNA sequences

Replicate	Scaled by μ				Converted into demographic units			
	Parameter	High point	HPD95		Parameter	High point	HPD95	
			Low	High			Low	High
A	θ_{ENP}	42.7	24.7	72.5	$N_{\mathrm{e(ENP)}}$	28,230	16,330	47,932
B	θ_{ENP}	43.1	24.7	73.5	$N_{\mathrm{e(ENP)}}$	28,495	16,330	48,593
C	θ_{ENP}	42.7	24.7	73.5	$N_{\mathrm{e(ENP)}}$	28,230	16,330	48,593
A2	θ_{ENP}	42.9	24.9	72.5	$N_{\mathrm{e(ENP)}}$	28,363	16,462	47,932
A	θ_{GC}	0.5	0	3.5	$N_{\mathrm{e(GC)}}$	331	0	2,314
B	θ_{GC}	0.3	0	3.5	$N_{\mathrm{e(GC)}}$	198	0	2,314
C	θ_{GC}	0.5	0	3.5	$N_{\mathrm{e(GC)}}$	331	0	2,314
A2	θ_{GC}	0.5	0	3.5	$N_{\mathrm{e(GC)}}$	331	0	2,314
A	θ_A	32.3	0	189.5	$N_{\mathrm{e(A)}}$	21,355	0	125,284
B	θ_A	32.7	0	189.5	$N_{\mathrm{e(A)}}$	21,619	0	125,284
C	θ_A	32.3	0	189.5	$N_{\mathrm{e(A)}}$	20,693	0	125,284
A2	θ_A	32.9	0	189.5	$N_{\mathrm{e(A)}}$	21,751	0	125,284
A	$m_{(ENP\rightarrow GC)}$	0.54	0	13.11	$N_{e(GC)}m_{(ENP\rightarrow GC)}$	0.83	0.04	3.14
B	$m_{(ENP\rightarrow GC)}$	0.61	0	15.28	$N_{e(GC)}m_{(ENP\rightarrow GC)}$	0.87	0.03	3.2
C	$m_{(ENP\rightarrow GC)}$	0.58	0	16.47	$N_{e(GC)}m_{(ENP\rightarrow GC)}$	0.83	0.03	3.38
A2	$m_{(ENP\rightarrow GC)}$	0.58	0	12.48	$N_{e(GC)}m_{(ENP\rightarrow GC)}$	0.89	0.04	3.13
A	$m_{(GC\rightarrow ENP)}$	2.15	0.58	4.53	$N_{e(ENP)}m(_{GC\rightarrow ENP})$	42.9	8.58	125.3
B	$m_{(GC\rightarrow ENP)}$	2.18	0.58	4.67	$N_{e(ENP)}m(_{GC\rightarrow ENP})$	47.18	8.74	134.5
C	$m_{(GC\rightarrow ENP)}$	2.15	0.54	4.81	$N_{e(ENP)}m(_{GC\rightarrow ENP})$	43.55	8.71	137.6
A2	$m_{(GC\rightarrow ENP)}$	2.15	0.58	4.53	$N_{e(ENP)}m(_{GC\rightarrow ENP})$	43.64	8.73	127.4
A	t	0.02	0	39.98	T	1370	0	2,738,356
B	t	0.02	0	39.98	T	1370	0	2,738,356
C	t	0.02	0	39.98	T	1370	0	2,738,356
A2	t	0.02	0	39.98	T	1370	0	2,738,356

Notes: Θ denotes: $4N_e\mu$, where N_e denotes the effective population size, and μ the generational mutation rate per locus. ENP, GC and A, denotes the eastern North Pacific, Gulf of California and ancestral population, respectively. The immigration rate scaled by the mutation rate is denoted by *m* and the immigration rate by $N_e m$, where the direction of immigration is denoted by →. The divergence time scaled by the mutation rate is denoted by *t* and the divergence time in years by *T*. Demographic conversions were based on a mutation rate of 5.2 x 10^{-8} per site per year and a generation time of 25.9 years. A, B and C denotes the different replicates using different seeds (21062018, 7252018234 and 235725). A2 was started using the Markov chain state space of A.

Table S7. IMA2P estimates based upon mtCR DNA sequences and microsatellite genotypes

Replicate	Scaled by μ				Converted into demographic units			
	Parameter	High point	HPD95 Low	HPD95 High	Parameter	High point	HPD95 Low	HPD95 High
A	θ_{ENP}	19.3	8.1	173.7	$N_{\mathrm{e(ENP)}}$	7,188	3,017	64,696
B	θ_{ENP}	19.3	7.7	168.5	$N_{\mathrm{e(ENP)}}$	7,188	2,868	62,759
C	θ_{ENP}	19.3	8.3	172.3	$N_{\mathrm{e(ENP)}}$	7,188	3,091	64,174
D	θ_{ENP}	19.1	7.9	169.7	$N_{\mathrm{e(ENP)}}$	7,114	2,942	63,206
A2	θ_{ENP}	17.5	6.1	163.3	$N_{\mathrm{e(ENP)}}$	6,518	2,272	60,822
B2	θ_{ENP}	18.7	8.9	177.5	$N_{\mathrm{e(ENP)}}$	6,965	3,315	66,111
A	θ_{GC}	0.1	0	1.7	$N_{\mathrm{e(GC)}}$	37.25	0	633.2
B	θ_{GC}	0.1	0	1.9	$N_{\mathrm{e(GC)}}$	37.25	0	707.7
C	θ_{GC}	0.1	0	1.7	$N_{\mathrm{e(GC)}}$	37.25	0	633.2
D	θ_{GC}	0.1	0	1.7	$N_{\mathrm{e(GC)}}$	37.25	0	633.2
A2	θ_{GC}	0.1	0	1.9	$N_{\mathrm{e(GC)}}$	37.25	0	707.7
B2	θ_{GC}	0.1	0	1.7	$N_{\mathrm{e(GC)}}$	37.25	0	633.2
A	θ_A	21.5	11.9	61.3	$N_{\mathrm{e(A)}}$	8,008	4,432	22,832
B	θ_A	21.3	11.3	75.3	$N_{\mathrm{e(A)}}$	7,933	4,209	28,046
C	θ_A	21.5	11.9	67.5	$N_{\mathrm{e(A)}}$	8,008	4,432	25,141
D	θ_A	21.5	11.9	72.5	$N_{\mathrm{e(A)}}$	8,008	4,432	27,003
A2	θ_A	19.9	13.10	34.10	$N_{\mathrm{e(A)}}$	7,412	4,879	12,701
B2	θ_A	21.7	12.1	59.9	$N_{\mathrm{e(A)}}$	8,082	4,507	22,310
A	$m_{(ENP\rightarrow GC)}$	6.63	0.57	45.39	$N_{e(GC)}m_{(ENP\rightarrow GC)}$	3	0	51
B	$m_{(ENP\rightarrow GC)}$	5.73	0.33	44.43	$N_{e(GC)}m_{(ENP\rightarrow GC)}$	3	0	51
C	$m_{(ENP\rightarrow GC)}$	6.15	0.51	57.03	$N_{e(GC)}m_{(ENP\rightarrow GC)}$	3	0	51
D	$m_{(ENP\rightarrow GC)}$	6.03	0.33	41.01	$N_{e(GC)}m_{(ENP\rightarrow GC)}$	3	0	51
A2	$m_{(ENP\rightarrow GC)}$	3.57	0.09	29.79	$N_{e(GC)}m_{(ENP\rightarrow GC)}$	3	0	51
B2	$m_{(ENP\rightarrow GC)}$	5.19	1.29	48.69	$N_{e(GC)}m_{(ENP\rightarrow GC)}$	3	0	51
A	$m_{(GC\rightarrow ENP)}$	0.09	0	5.85	$N_{e(ENP)}m_{(GC\rightarrow ENP)}$	1.35	0	339.7
B	$m_{(GC\rightarrow ENP)}$	0.21	0	5.37	$N_{e(ENP)}m_{(GC\rightarrow ENP)}$	1.21	0	313.5
C	$m_{(GC\rightarrow ENP)}$	0.03	0	5.37	$N_{e(ENP)}m_{(GC\rightarrow ENP)}$	1.43	0	330.2
D	$m_{(GC\rightarrow ENP)}$	1.77	0	5.13	$N_{e(ENP)}m_{(GC\rightarrow ENP)}$	1.31	0	288.1
A2	$m_{(GC\rightarrow ENP)}$	0.69	0	3.87	$N_{e(ENP)}m_{(GC\rightarrow ENP)}$	0.96	0	198.4
B2	$m_{(GC\rightarrow ENP)}$	2.55	0	5.37	$N_{e(ENP)}m_{(GC\rightarrow ENP)}$	1.57	0	335.8
A	t	0.1	0	10.66	T	3859	0	411,334
B	t	0.06	0	15.18	T	2315	0	585,745
C	t	0.1	0	10.02	T	3859	0	386,638
D	t	0.1	0	7.38	T	3859	0	284,769
A2	t	0.06	0	1.62	T	2,315	0	62,510
B2	t	0.14	0	7.66	T	5,402	0	295,574

Notes: Θ denotes: $4N_e\mu$, where N_e denotes the effective population size, and μ the generational mutation rate per locus. ENP, GC and A, denotes the eastern North Pacific, Gulf of California and ancestral population, respectively. The immigration rate scaled by the mutation rate is denoted by m and the immigration rate by $N_e m$, where the direction of immigration is denoted by →. The divergence time scaled by the mutation rate is denoted by t and the divergence time in years by T. Demographic conversions were based on a mutation rate of 5.2 x 10^{-8} per site per year and a generation time of 25.9 years in case of the mtCR DNA sequences and transformed to the per locus mutation rate. The mutation rate employed for the microsatellite genotypes was at 7 x 10^{-4} per generation (the estimate from MSVAR). A, B, C and D denotes the different replicates using different random seeds (1212519, 9102521, 216222 and 410272). A2 and B2 were started using the Markov chain state space of the previous run A and B respectively. A2 was the run with the highest ESS. $N_{e(GC)}m_{(ENP\rightarrow GC)}$ was not estimated (see Figure S8) but the migration rate scaled by the mutation rate was (m, Figure S9).

Table S8. DIYABC model estimation and checking

Summary statistics	Observed value	Recent split	Older split
ENP mean number of alleles*	10.7778	0.506	0.5375
GC mean number of alleles*	7.6111	0.1685	0.8305
ENP mean genic diversity*	0.8242	0.3795	0.42
GC mean genic diversity*	0.4926	0.001(***)	0.017(*)
ENP mean size variance*	12.0047	0.3	0.27
GC mean size variance*	7.3355	0.083	0.187
ENP mean Garza-Williamson's M *	0.8308	0.655	0.733
GC mean Garza-Williamson's M*	0.6524	0.2295	0.721
ENP&GC mean number of alleles	12.0556	0.525	0.615
ENP&GC mean genic diversity	0.5446	0.001(***)	0.0165(*)
ENP&GC mean size variance	8.3625	0.126	0.212
ENP&GC FST*	0.2192	0.999(***)	0.9655(*)
ENP&GC Classification index *	3.1082	0.973(*)	0.426
GC&NP Classification index *	1.6993	0.373	0.249
ENP&GC Shared allele distance	0.1991	0.837	0.826
ENP&GC $(d\mu)^2$ distance*	7.9763	0.996(**)	0.875
ENP number of haplotypes*	8	0.327	0.2655
GC number of haplotypes*	4	0.32	0.7435
ENP number of segregating sites*	8	0.4665	0.547
GC number of segregating sites*	5	0.282	0.671
ENP mean of pairwise differences*	1.5533	0.1065	0.1045
GC mean of pairwise differences*	0.1668	0.019(*)	0.203
ENP variance of pairwise differences	1.7864	0.177	0.1985
GC variance of pairwise differences	0.1933	0.021(*)	0.223
ENP Tajima's D*	-0.8472	0.0295(*)	0.024(*)
GC Tajima's D*	-1.4811	0(***)	0.007(**)
ENP private segregating sites*	6	0.978(*)	0.775
GC private segregating sites*	3	0.992(**)	0.9965(**)
ENP mean number of the rarest nucleotide at segregating sites	3.25	0.2005	0.227
GC mean number of the rarest nucleotide at segregating sites	4.4	0.014(*)	0.154
ENP variance of number of the rarest nucleotide at segregating sites	12.4375	0.6885	0.715
GC variance of number of the rarest nucleotide at segregating sites	34.24	0.108	0.477
ENP&GC number of haplotypes	11	0.529	0.468
ENP&GC number of segregating sites	11	0.949	0.965(*)
ENP&GC mean of pairwise differences (W)*	0.1799	0.019(*)	0.196
ENP&GC mean of pairwise differences (B)	1.1254	0.0555	0.059
ENP&GC FST*	0.8402	0.981(*)	0.715

Notes: For each individual summary statistic, the value of the observed dataset is presented as well as the proportion of datasets (1,000 datasets simulated with values drawn from the posterior distributions) with values lower than the observed value. Upper part of table contains the summary statistics employed for microsatellite genotypes. Lower part of the table lists the summary statistics employed for the mtCR DNA sequences. The summary statistics with an asterisk were used for the estimation. All summary statistics were employed in the model checking.

Figures

Figure S1. Gelman-Rubin plots of the 11 BAYESASS estimations

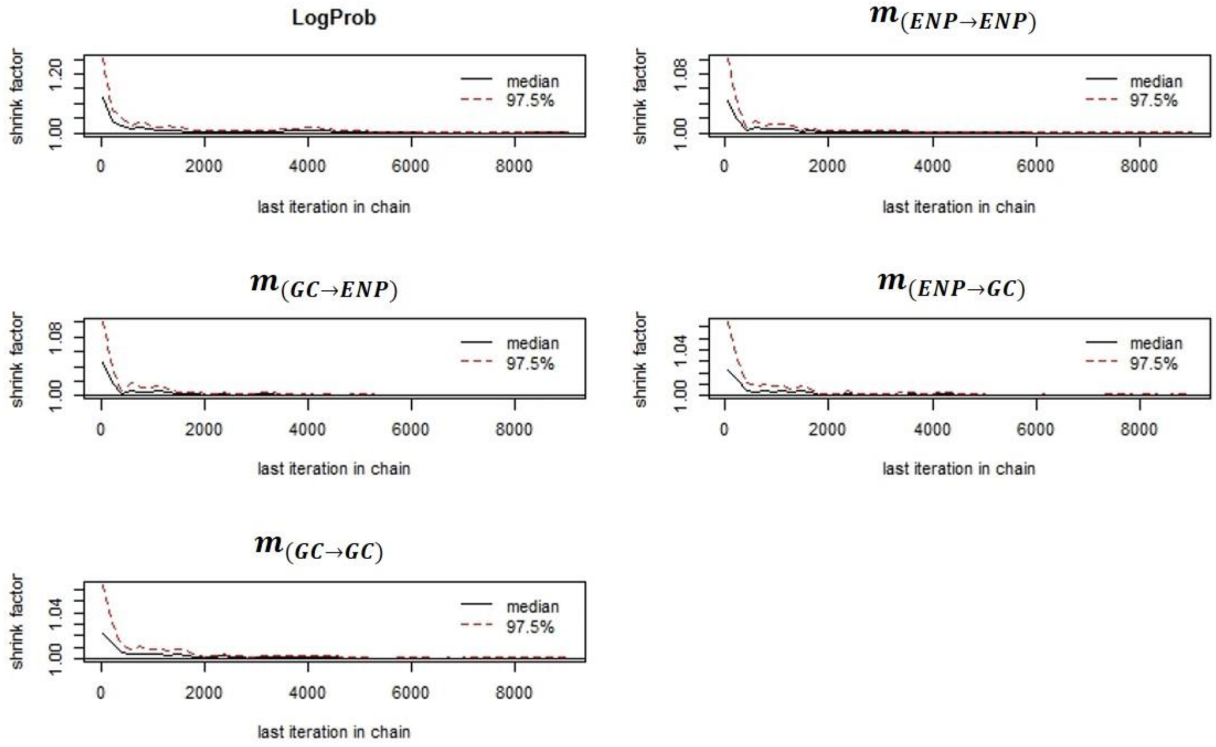

Note: The Gelman-Rubin diagnostic was estimated at 1.

Figure S2. Posterior distributions of contemporary migration rates per generation (m) estimated with BAYESASS

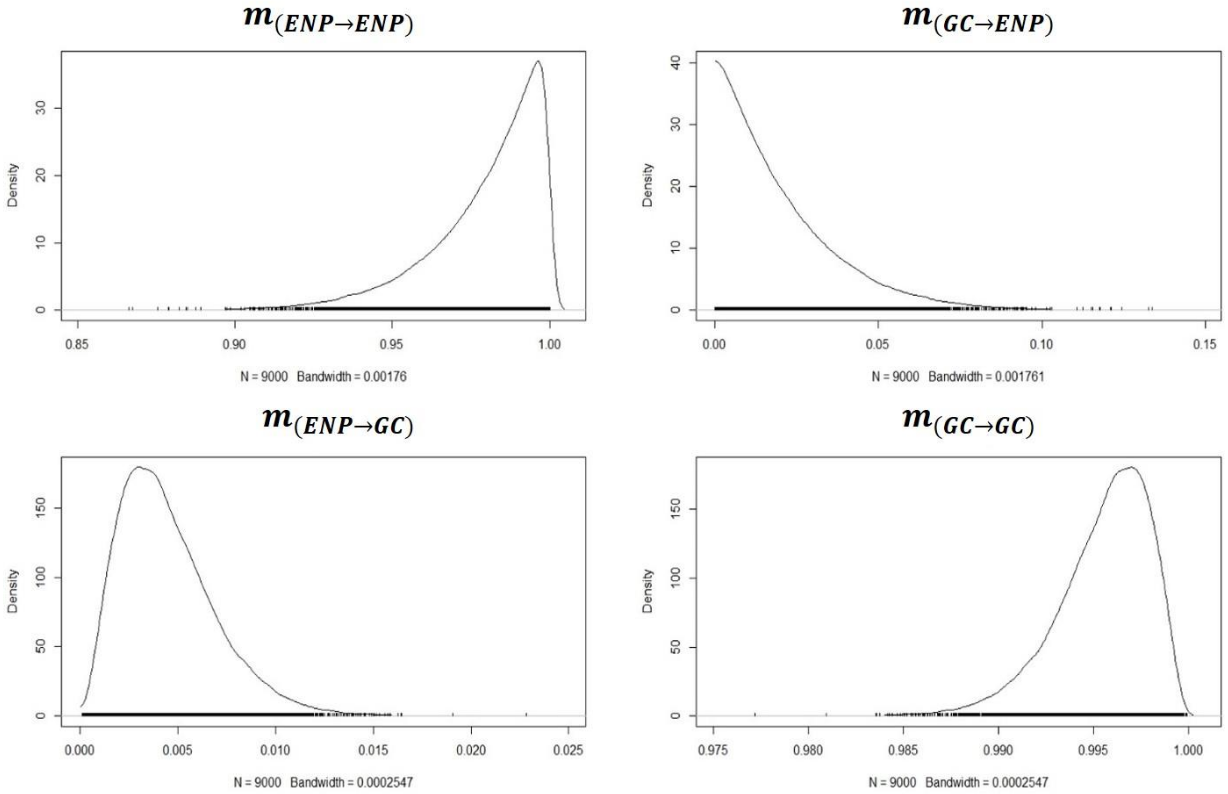

Notes: The arrows indicate the direction of the immigrants. For example, $m_{(ENP \to GC)}$ denotes the fraction of individuals in the Gulf of California (GC) that are immigrants from the eastern North Pacific (ENP).

Figure S3. MSVAR-based estimates of the ancestral and current effective population size, and time of population size change

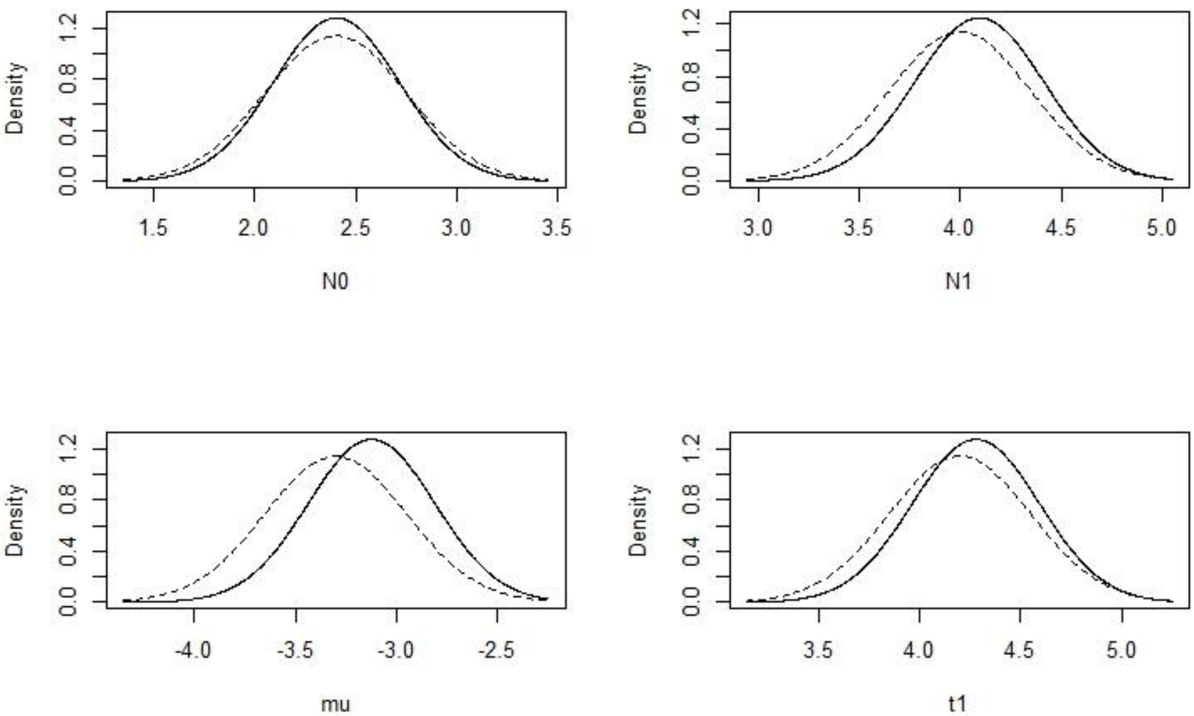

Notes: Prior (broken line) and posterior distribution (continuous line) of the current population size, N_0; the ancestral population size, N_1; the mutation rate, mu, and the time of population size change, t_1.

Figure S4. Gelman-Rubin plots for the two MSVAR-based estimations

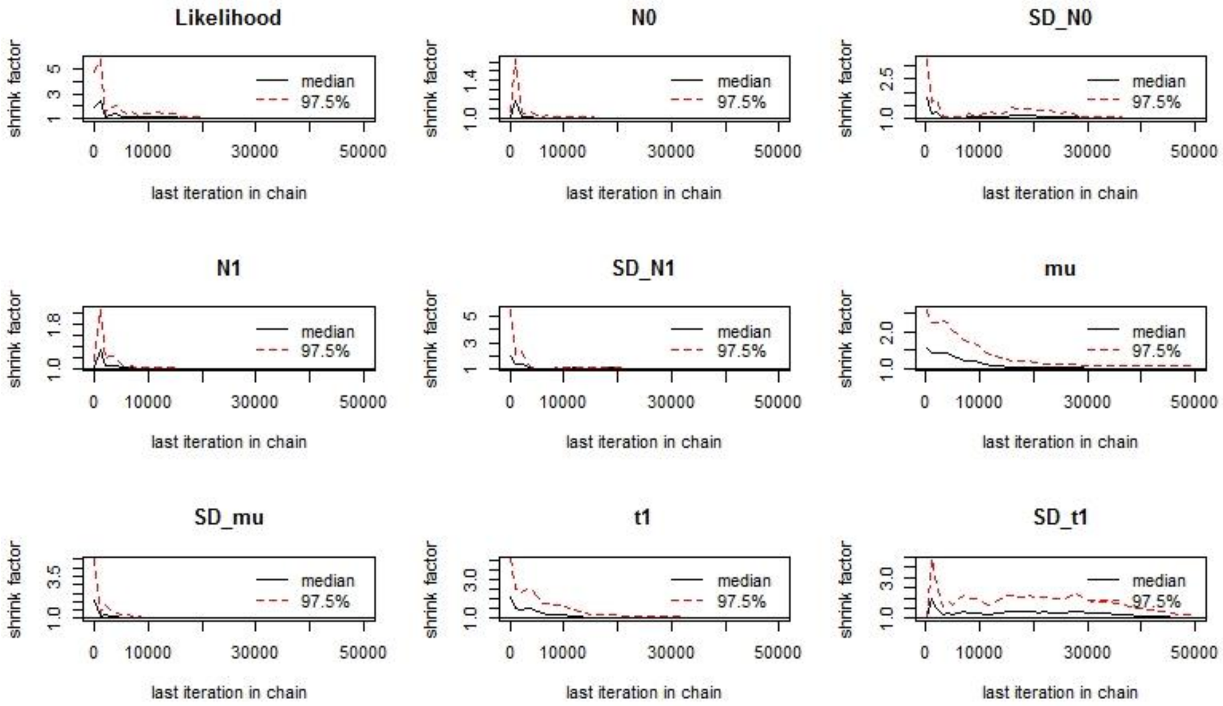

Note: The Gelman-Rubin diagnostic was estimated at 1.09. N_0, denotes the current population size; N_1, the ancestral population size; mu, the mutation rate; t_1, the time of the change in population size and SD the standard deviation.

Figure S5. IMA2P posterior distributions of N_e and T estimated from mtCR DNA sequences only.

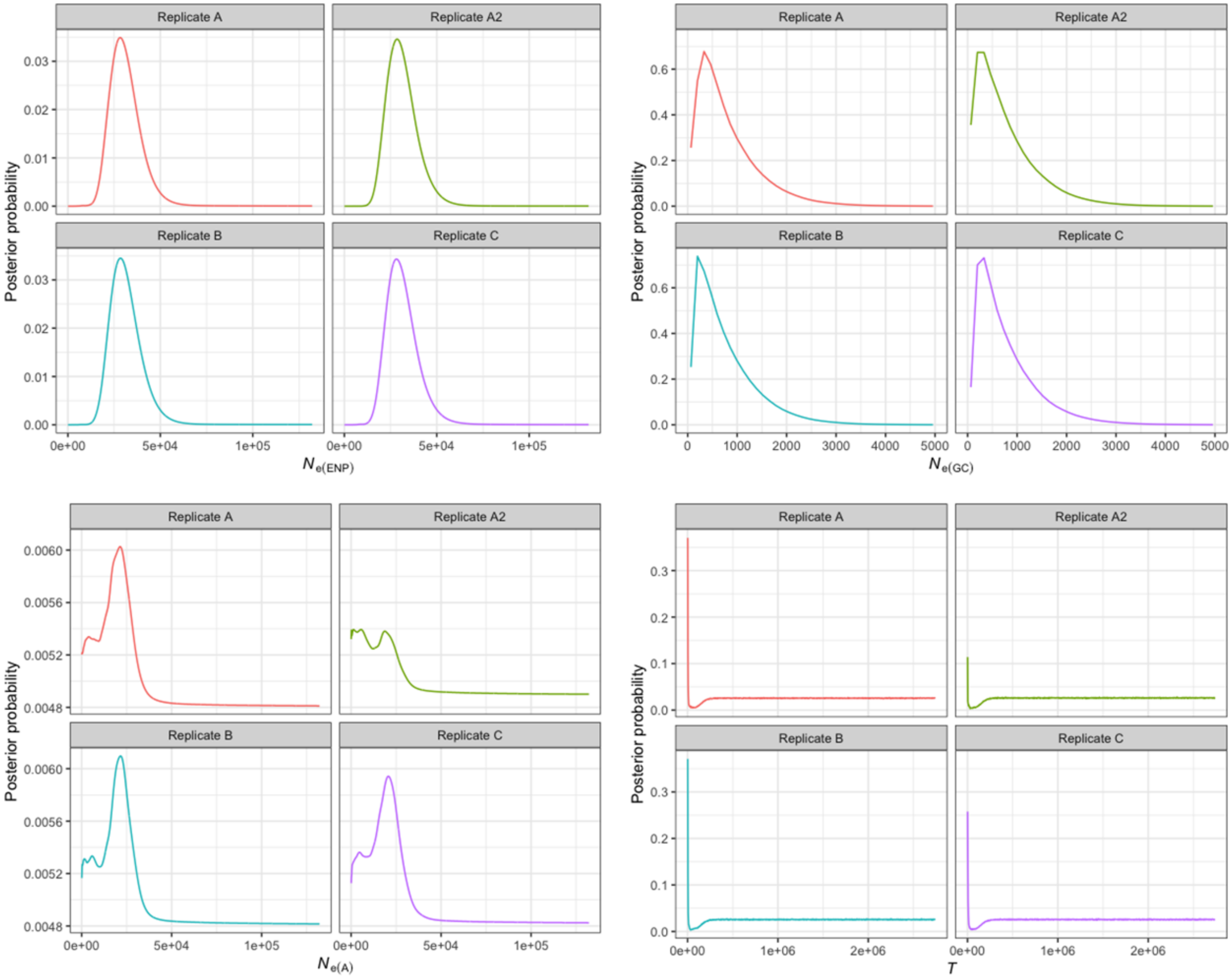

Notes: N_e, denotes the effective population size; T, divergence time. ENP, GC and A, denotes the eastern North Pacific, Gulf of California and ancestral population, respectively.

Figure S6. IMA2P posterior distributions of N_e and T based upon combined mtCR DNA sequences and microsatellite genotypes.

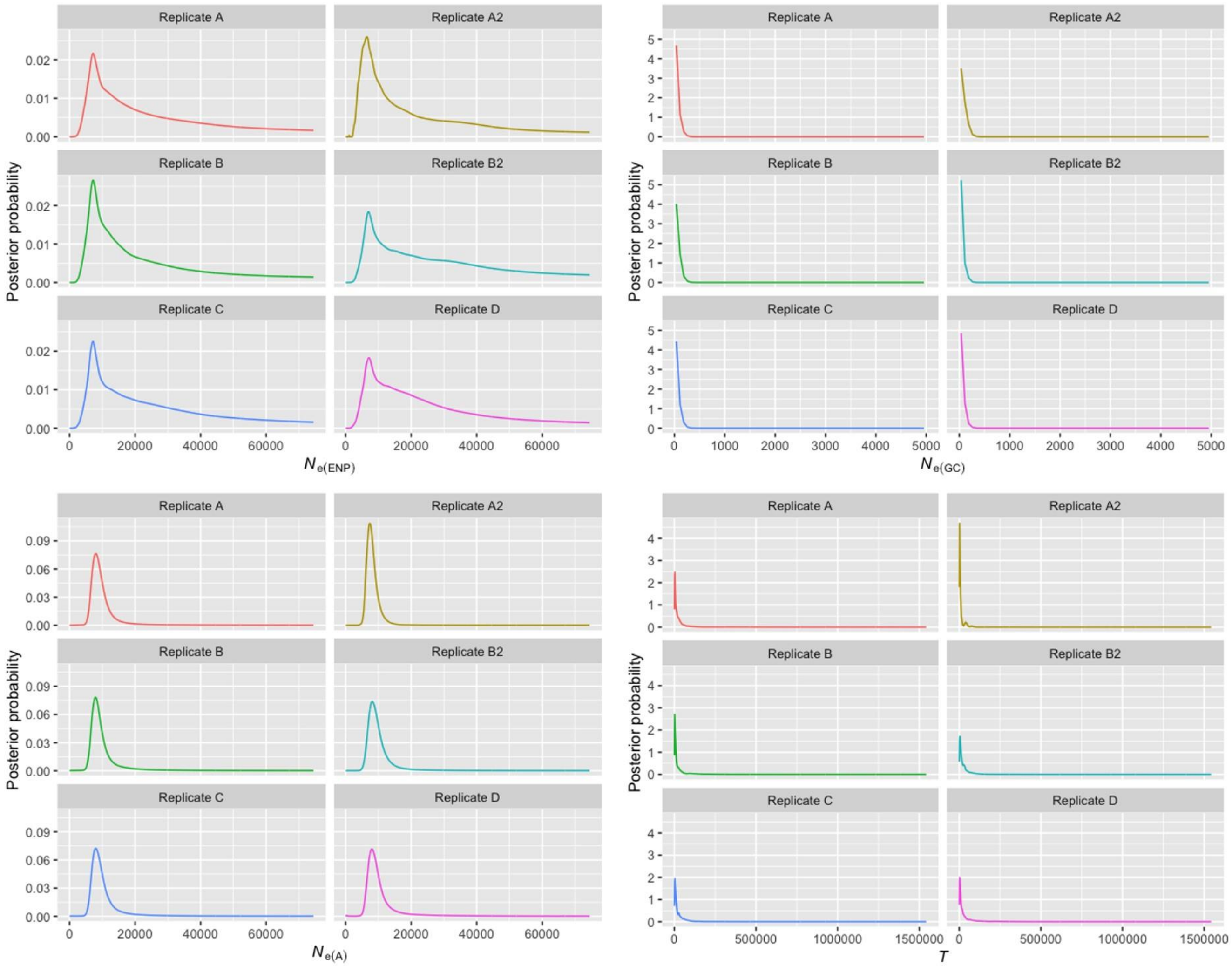

Notes: N_e denotes the effective population size and T the divergence time. ENP, GC and A, denotes the eastern North Pacific, Gulf of California and ancestral population, respectively.

Figure S7. IMA2P posterior distributions of $N_e m$ from mtCR DNA sequences only

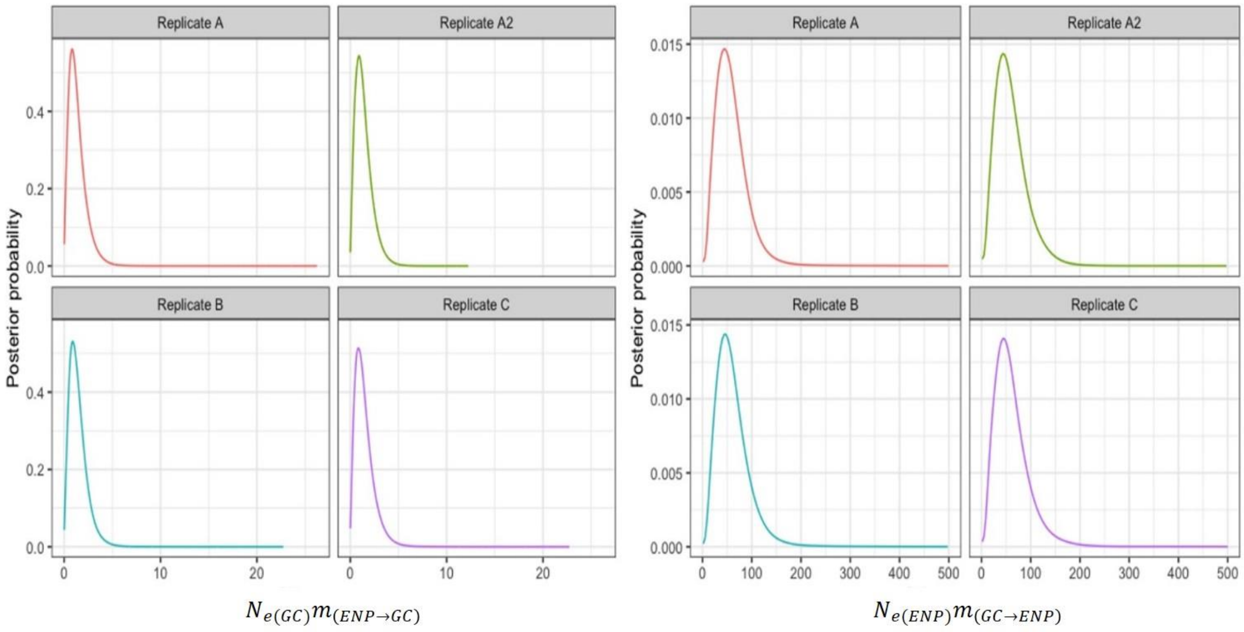

Notes: $N_e m$, denotes the population immigration rate; →, the direction of immigration, ENP, the eastern North Pacific and GC the Gulf of California.

Figure S8. IMA2P posterior distributions of $N_e m$ estimated from mtCR DNA sequences and microsatellite genotypes combined

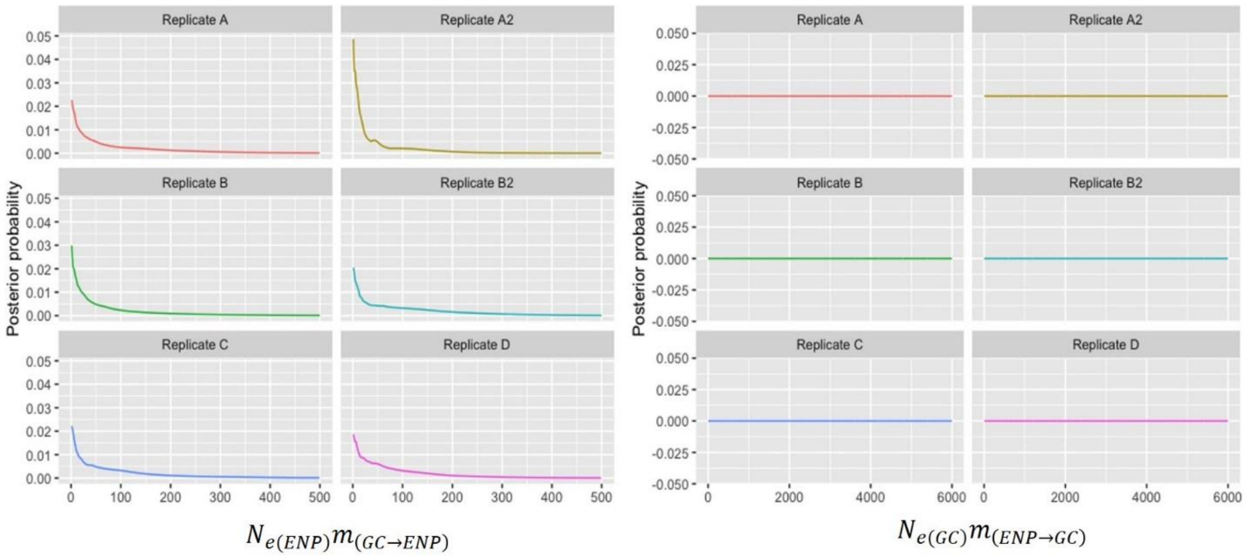

Notes: $N_e m$, denotes the immigration rate; →, the direction of immigration; ENP, the eastern North Pacific, and GC, the Gulf of California. $N_{e(GC)}m_{(ENP\rightarrow GC)}$ was not estimated but $m_{(ENP\rightarrow GC)}$ was (see Figure S9).

Figure S9. IMA2P posterior distributions of the immigration rate scaled by the mutation rate using mtCR DNA sequences and microsatellite genotypes

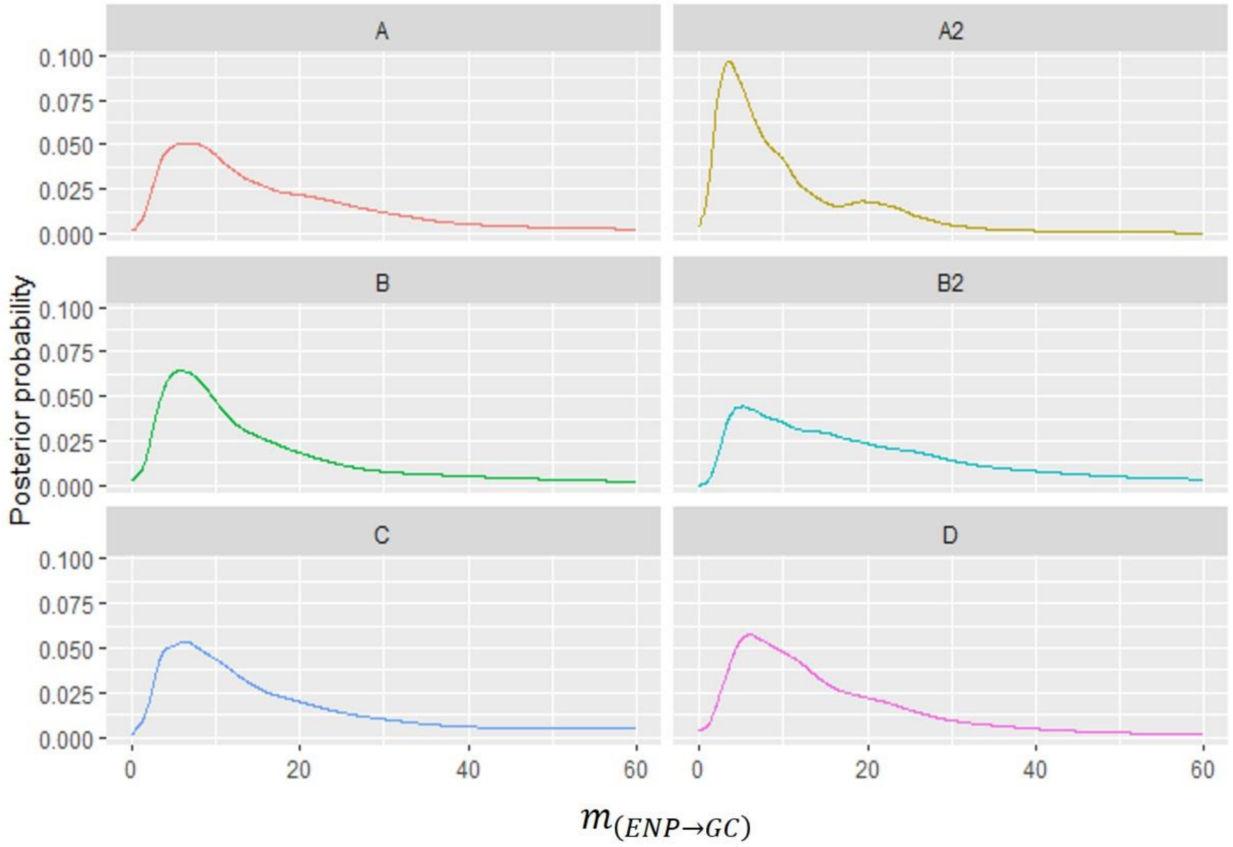

Notes: The direction of immigration is denoted by →; *m* denotes the immigration rate scaled by the mutation rate; ENP, the eastern North Pacific and GC the Gulf of California.

Figure S10. Logistic regression of posterior probabilities of the two scenarios tested in DIYABC and model checking procedure using a principal component analysis of summary statistics.

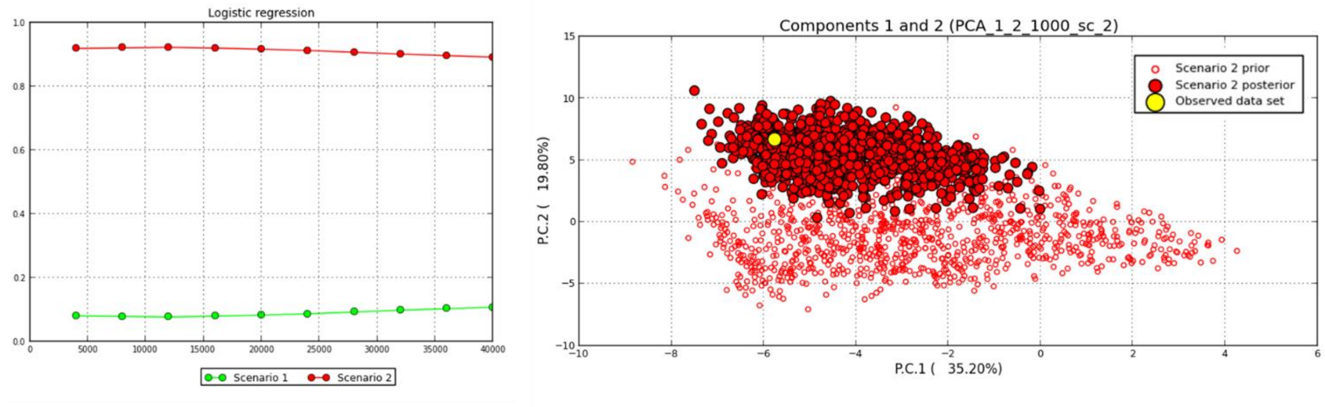

Notes: The best supported scenario was the one with an older population divergence time (> 1 kya; scenario 2). The open red circles represent the datasets simulated from the prior distribution of the parameters, the filled red circles are from the posterior distribution and the yellow circle represents the observed dataset.

Figure S11. DIYABC estimates, prior and posterior distributions for the scenario with an older population divergence time (scenario 2).

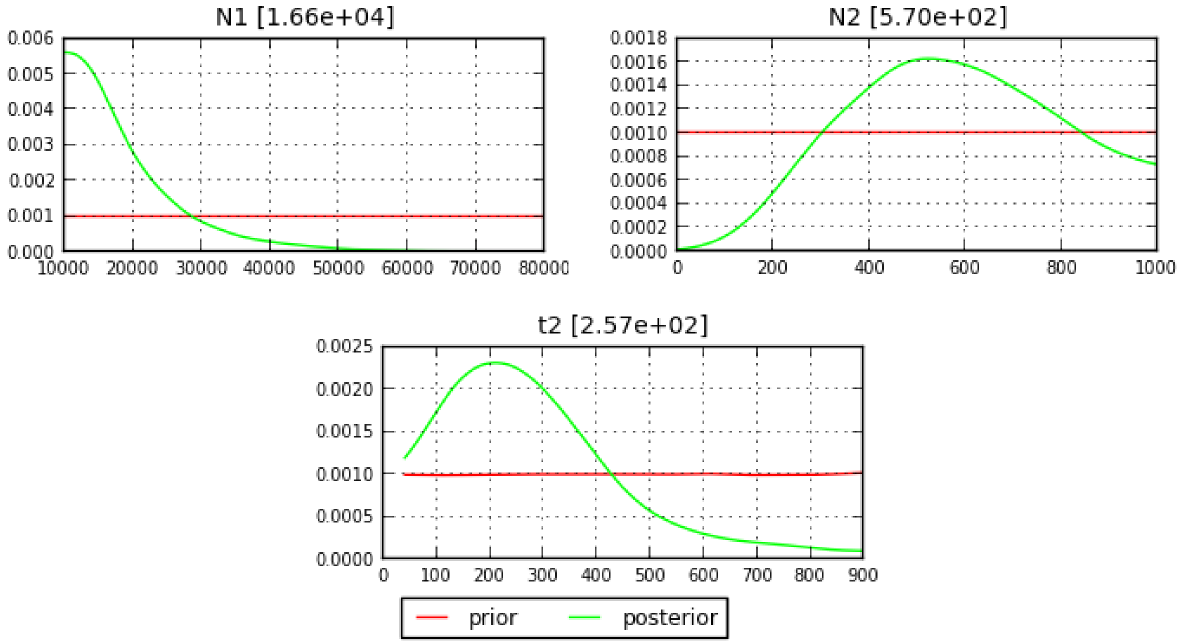

Notes: N1, denotes the effective population size of the eastern North Pacific (mode: 11,800 [95% CI: 10,300 – 44,000]); N2, the effective population size of the Gulf of California (497 [95%CI: 195 - 963]) and t2, the population divergence (239 [95%CI: 68 - 732] generations, which is ≈6.2 [95%CI: 1.8 – 19] kya). The value next to the parameter in the plots is the median. The scenario only takes into account population divergence but not reductions in population size, duration of the change in population size nor migration between populations, so the parameter estimates might not be accurate.
